# A metabolic and physiological design study of *Pseudomonas putida* KT2440 capable of anaerobic respiration

**DOI:** 10.1186/s12866-020-02058-1

**Published:** 2021-01-06

**Authors:** Linde F. C. Kampers, Jasper J. Koehorst, Ruben J. A. van Heck, Maria Suarez-Diez, Alfons J. M. Stams, Peter J. Schaap

**Affiliations:** 1 Laboratory of Systems and Synthetic Biology, Wageningen University and Research Centre, Stippeneng 4, 6708 WE Wageningen, The Netherlands; 2 Laboratory of Microbiology, Wageningen University and Research Centre, Stippeneng 4, 6708 WE Wageningen, The Netherlands

**Keywords:** *Pseudomonas*, Anaerobic respiration, Anaerobic fermentation, Computational design, Bioinformatics, Microbial lifestyle engineering

## Abstract

**Background:**

*Pseudomonas putida* KT2440 is a metabolically versatile, HV1-certified, genetically accessible, and thus interesting microbial chassis for biotechnological applications. However, its obligate aerobic nature hampers production of oxygen sensitive products and drives up costs in large scale fermentation. The inability to perform anaerobic fermentation has been attributed to insufficient ATP production and an inability to produce pyrimidines under these conditions. Addressing these bottlenecks enabled growth under micro-oxic conditions but does not lead to growth or survival under anoxic conditions.

**Results:**

Here, a data-driven approach was used to develop a rational design for a *P. putida* KT2440 derivative strain capable of anaerobic respiration. To come to the design, data derived from a genome comparison of 1628 *Pseudomonas* strains was combined with genome-scale metabolic modelling simulations and a transcriptome dataset of 47 samples representing 14 environmental conditions from the facultative anaerobe *Pseudomonas aeruginosa*.

**Conclusions:**

The results indicate that the implementation of anaerobic respiration in *P. putida* KT2440 would require at least 49 additional genes of known function, at least 8 genes encoding proteins of unknown function, and 3 externally added vitamins.

## Background

*Pseudomonas putida* KT2440 is a HV1-certified [1], genetically accessible [2–7] and metabolically versatile [8, 9] species, which makes it an interesting adaptable industrial workhorse [10–12]. However, its strict aerobic lifestyle is an industrial disadvantage [4, 13–16] as the strict requirement for dissolved O2 results in increased costs of large-scale cultivation and may lead to unstable production rates due to inadequate local oxygen supply caused by oxygen fluctuations. Its strict aerobic nature also excludes production of O2-sensitive enzymes, pathway intermediates or target products.

Most *Pseudomonas* species are facultative anaerobes and use an inorganic compound such as nitrate as alternate terminal electron receptor. This includes species closely related to the *P. putida* KT2440 strain, such as *P. fluorescens* and *P. denitrificans*. Only one *Pseudomonas* species is capable of anaerobic fermentation: *Pseudomonas aeruginosa* [17–20]. *P. aeruginosa* is capable of arginine fermentation and pyruvate fermentation, although the latter only leads to prolonged survival under anoxic conditions, not to growth [18–20].

As there is a relatively short evolutionary distance between the strict aerobic *P. putida* KT2440 and facultative anaerobic *Pseudomonas* species compared to other anaerobic bacteria [21], it could be reasoned that a minimal set of adaptations would be required to change an aerobic *Pseudomonas* species into an facultative anaerobic one. Through the implementation of a rational engineering cycle, this strain could be adapted to a facultative anaerobic lifestyle. In an attempt to obtain a *P. putida* KT2440 derived strain capable of anaerobic fermentation a Design, Build, Test, Learn-engineering cycle [22] was performed in earlier work [23] to obtain an *P. putida* KT2440 strain capable of anaerobic fermentation. Using genome metabolic models (GSMs) iJP962 and iJP746 combined with a protein domain comparison (PDC) between six aerobic *Pseudomonas putida* strains including KT2440 and six facultative anaerobic *Pseudomonas* strains, three key enzymes were selected and included in the final design: acetate kinase (encoded by *ackA*), dihydroorotate dehydrogenase (*pyrK-pyrD B*) and ribonucleotide triphosphate reductase class III (*nrdD-nrdG*). This design was built, and the resulting recombinant strain showed growth under micro-oxic conditions [23]. Earlier work already described an increase in survival rates upon introduction of solely acetate kinase [4, 14], and since the model predictions used in the design only considered full anoxic conditions, survival rates of the recombinant strains under anoxic conditions needed to be tested.

Here, we (i) determined the survival rates of the previously constructed recombinant strains under anoxic conditions, (ii) identified limitations for anaerobic growth through respiration, and (iii) composed a new design for a recombinant *P. putida* KT2440 capable of anaerobic respiration. In pursuit of this goal we expanded upon earlier work using the current wealth of genome data available on *P. putida* and other *Pseudomonas* species by inclusion of 1628 strains in an extensive comparison of the protein domain content [24]. Random forest, a machine learning method, was used to identify key protein domains associated with “anaerobic growth”. Transcriptome data of the *Pseudomonas aeruginosa* type strain PA14 cultures grown in 14 different conditions [25] were also considered and integrated with previous and newly obtained GSM simulation results to compose a final design.

## Methods

### Bacterial strains and cultivation conditions

Bacterial strains and plasmids are listed in in Table S1. For plasmid construction see previous work [23]. *E. coli* CC118λpir was used for cloning procedures and plasmid maintenance, and was routinely cultivated at 37 °C in aerated conditions in LB medium (10 g/l tryptone, 10 g/l NaCl and 5 g/l yeast extract), optionally containing antibiotics for selection (50 μg/ml kanamycin or 50 μg/ml ampicillin). For solid medium, 15 g/l agar was added to the medium. *P. putida* KT2440 was routinely cultivated under oxic conditions at 30 °C in LB medium. Experiments were performed in De Bont minimal medium [26] (3.88 g/l K2HPO4, 1.63 g/l NaH2PO4· 2H2O, 2.00 g/l (NH4)2SO4, 0.1 g/l MgCl2·6H2O, 10 mg/l EDTA, 2 mg/l ZnSO4· 7H2O, 1 mg/l CaCl2 · 2H2O, 5 mg/l FeSO4· 7H2O, 0.2 mg/l Na2MoO4·2H2O, 0.2 mg/l CuSO4·5H2O, 0.4 mg/l CoCl2·6H2O, 1 mg/l MnCl2·2H2O), with 20 g/l gluconic acid as the sole carbon source. In previous work, different carbon sources were tested for optimal performance [23]. Gluconic acid was used for optimal growth by eliminating ATP consumption for substrate uptake due to passive membrane transport. The medium was supplemented with 50 μg/ml kanamycin when indicated. Precultures were prepared aerobically overnight at 200 rpm at 30 °C.

### Anoxic survival experiment

Oxygen gradients served to allow the recombinant strains to grow in micro-oxic conditions as described in [23]. Anoxic cultivation of *P. putida* KT2440 recombinants unpassed or passed over oxygen gradients was performed at 30 °C in 50 ml glass 20 mm aluminium crimp cap vials with rubber stoppers (Glasgerätebau Ochs Laborfachhandel e.K.) in 30 ml DeBont with 20 g/l gluconic acid, 1 mg/l resazurin and 50 μg/ml kanamycin as selection marker for recombinant strains. Where indicated, a 1000x diluted vitamin mix was added (0.02 g/l biotin, 0.2 g/l nicotinamide, 0.1 g/l p-aminobenzoic acid, 0.2 g/l thiamin, 0.1 g/l pantothenic acid, 0.5 g/l pyridoxamine, g/l cyanocobalamin, 0.1 g/l riboflavin). Before inoculation, the vials were gas exchanged with CO2/N2. Inoculation was done with aerobically pre-cultured bacterial sample at an OD600 of 0.05. Approx. 8 h after inoculation, the resazurin became completely colourless, indicating obtainment of anaerobic conditions. Samples were taken using sterile CO2 flushed 1.5″ needles (BD Microlance) and 3–5 ml syringes (ThermoFisher) to avoid O2 exposure. Anoxic conditions were ensured as the resazurin turned from colourless to bright pink within seconds in extracted samples. Survival rates were analysed by colony forming units (CFU) determination. A dilution series was made and five drops of 10 μl per dilution were applied onto LB-agar plates without selection marker, which were incubated o/n at 30 °C. Colonies were counted manually, and photos were taken of the plates. Gram-staining was performed as an intermediate check of culture purity, according to manufacturers’ instructions (Gram-staining kit Machery-Nagel, Germany), while genome sequencing was applied to ensure culture purity at the start and end of every experiment.

### Statistical analysis

Experiments were independently repeated six times with biological triplicates in each separate experiment. Figures represent the mean values of corresponding biological triplicates and the standard deviation. The level of significance of the differences when comparing results was evaluated by means of analysis of variance (ANOVA), with α = 0.05.

### Genome annotation

Information on the oxygen requirements of 16,989 *Pseudomonas* strains was obtained from the Gold database [27]. Per species, extensive literature research was performed to validate their aerobicity (Data S5). One thousand six hundred twenty-eight Genomes of facultative anaerobic and strict anaerobic strains from the *Pseudomonas* genus were obtained from the European Nucleotide Archive repository in March 2015 [28]. All genomes were de-novo annotated in SAPP [29] using Prodigal for gene prediction (version 2.6) [30], 2010] and InterProScan version 5.4–47.0 [31] for functional annotation using Pfam [32].

### Comparisons of protein domain content

The positions (start and end on the protein sequence) of the protein domains and their order in a protein when multiple domains were present, were used to identify domain architecture (i.e. combinations of protein domains). Protein domain architectures were labelled by the ordered list of Pfam identifiers as described in [33]. Protein domain architectures identified in each genome sequence were stored in a matrix. From this a binarized domain architecture presence-absence matrix was extracted and used as input for principal component analysis using the standard R-package prcomp and hierarchical clustering using the standard R-package hclust.

### Gene persistence

The persistence of a gene in a taxonomic group or group of genomes can be defined as

$$ Persistence=\frac{N(orth)}{N} $$

where N (orth) is the number of genomes carrying a given ortholog and N is the number of genomes considered [24]. For the set of 1628 considered genomes. Orthologous genes were identified through identity of protein domain architectures considering copy number. Resulting protein domain contents were analysed through protein domain comparison (PDC).

### Feature selection using random forest

The random forest classification algorithm was used to classify the genome sequences in aerobic and facultative anaerobic species with the goal to identify the domains (features) responsible for the separation in these two groups (feature selection). Three hundred randomly selected genomes from aerobic and anaerobic *Pseudomonas* species were selected to train random forest models. The process was repeated one hundred times. The resulting 100 different models were used to weigh 5831 protein domains from both aerobic and anaerobic *Pseudomonas* species. Variable selection was used to identify the most influential domains for classification in aerobic and facultative anaerobic strains, yielding 100 Gini coefficients, representing the importance of a protein domain for separation per protein domain. Gini coefficients were combined into the cumulative Gini coefficient. The resulting protein domains were separated into aerobic/anaerobic specific protein domains before further analysis.

### Transcriptome data analysis

A publicly available *P. aeruginosa* transcriptome data set was retrieved from GEO database (accession number GSE55197) [25]. This dataset contains 47 samples corresponding to 14 environmental conditions, including changes in growth temperature, growth stage, osmolarity, concentration of ions in the media, and surface attachment and anaerobic respiration. For every gene, the log2 fold change of its expression values was calculated in comparing every possible condition with anaerobic respiration. Missing or infinity values arising from genes with very low counts in some condition(s) were imputed to 0 or ± 4, according to the significance of the differential expression (False discovery rate, fdr < 0.05). Normalization, fold change computations and differential expression analysis were performed using the R package DESeq [34].

### Genome-scale metabolic models

In this study we used the *P. putida* genome-scale metabolic models (GSMs) iJP962, iJN746 and iJN1411 [3, 5, 35]. iJN1411 was obtained directly from the authors [35]. GSM simulations were performed as described in [23], with uptake rates of up to 1000 mmol gdw^− 1^ h^− 1^ (gdw: grams dry weight) of copper, cobalt, iron, protons, water, sodium, nickel, ammonia, phosphate, sulphate, and nitrate, a maximal glucose uptake rate of 6.14 mmol gdw^− 1^ h^− 1^, based on experimentally measured uptake rates [36]. Thus, the in silico medium composition mimics the De Bont minimal medium used for the in vivo experiments.

## Results

### Insertion of acetate kinase in P. putida KT2440

Previous designs to obtain *P. putida* strains surviving anoxic conditions were conceptually based on the hypothesis that survival in anoxic conditions was prevented by a lack of energy conservation and redox balancing [4, 13–16]. Expression of the acetate kinase gene from *P. aeruginosa* and *E. coli* was reported to result in an extended survival under anoxic conditions [4, 14]. Expression of the acetate kinase gene (*ackA*) from *E. coli* combined with class I dihydroorotate hydrogenase (*pyrK-pyrD B*) and class III ribonucleotide triphosphate reductase (*nrdD-nrdG*) from *L. lactis* successfully led to growth under micro-oxic conditions [23].

To determine the tolerance to anoxic conditions of a *P. putida* KT2440 recombinant strain enriched with *ackA, pyrK-pyrD B* and *nrdD-nrdG* and of a negative control strain carrying an empty plasmid to anoxic conditions and to analyse the effect of an adaptation over oxygen gradients as performed earlier [23], an 18-day anoxic survival experiment was performed. After inoculation at a standardized cell density under oxic conditions, cultures were incubated overnight in capped gas-exchanged vials in oxygen-depleted medium (see Materials and Methods). To optimise growth gluconic acid was used as the main carbon source. In a previous design, gluconic acid was shown to offer better results compared to a series of other carbon sources [23]. The survival rate was determined by performing colony forming unit (CFU) counts at set time points over a period of 18 days, with T0 being the start of the experiment in anoxic conditions (supplementary Figures S1, S2, S3, S4, S5). The results showed that in anoxic conditions there is no significant difference in survival rates between the negative control and any of the recombinant strains tested (ANOVA α = 0.05). Under these conditions, only the positive control, *E. coli* BW25113 harbouring an empty plasmid, survived.

### Design requirements for a P. putida KT2440 derivative strain capable of anaerobic respiration

The failure of the previous, fermentative, design [23] to grow under anoxic conditions could be explained by the heavy reliance on the two state of art genome-scale models (GSMs) used in this design, which currently do not include an accurate representation of the complete redox balance and its intricate involvement in the metabolism. Additionally, while the protein domain comparison performed in the previous study showed apparent differences between aerobic and anaerobic strains in availability of protein domains, this analysis was performed on a limited set of strains.

Many facultative anaerobic *Pseudomonas* species are incapable of anaerobic fermentation, but rather perform anaerobic respiration. The close phylogenetic distances between some of these facultative anaerobic *Pseudomonas* species and *P. putida* KT2440 may suggest that acquiring a facultative anaerobic lifestyle via anaerobic respiration would require less genetic changes. To come to a rational design of *P. putida* KT2440 capable of anaerobic respiration, the previous methods were thus expanded upon by (i) using significantly more facultative anaerobic and aerobic *Pseudomonas* strains for domain analysis, (ii) inclusion of iJN1411, the latest metabolic reconstruction of *P. putida* KT2440 [35], and (iii) incorporation of an elaborate transcriptome analysis of anaerobic respiration of *P. aeruginosa* strains grown under anoxic conditions in comparison with 13 aerobic growth conditions [25]. Inclusion of such transcriptome data would show gene regulation due to growth under anoxic conditions, improving the design as it complements genome-based methods.

**Fig. 1:**
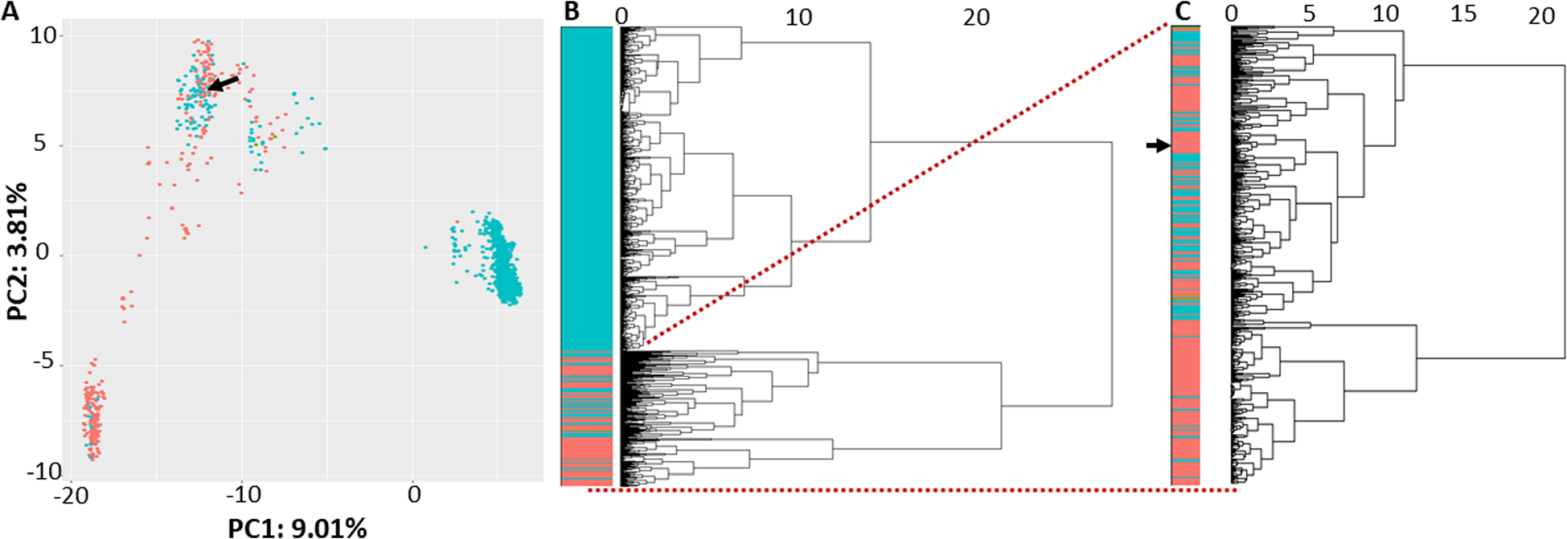
Protein domain content of 344 aerobic and 1284 facultative anaerobic *Pseudomonas* strains. Facultative anaerobic strains capable of respiration are indicated in blue, aerobic strains in red. **a** 2D Plot of PCA. Position of (*P. putida* KT2440 is marked with an arrow. Labels on the axes indicate fraction of the total variance explained by each component. **b** Observed distance tree based on presence/absence of protein domains. **c** Details of the main branch harbouring *P. putida* KT2440 (position indicated with an arrow). This branch consists of 138 anaerobic and 87 aerobic *Pseudomonas* species

For protein domain comparisons, the Pfam domain content of *P. putida* KT2440 was compared with 1627 other *Pseudomonas* strains with fully sequenced genomes. For each strain, a literature search was performed to determine oxygen requirements, yielding 344 obligate aerobic strains including KT2440 and 1284 facultative anaerobic strains. Strain specific differences in protein domain content were visualised using principal component analysis (PCA), and hierarchical clustering using domain presence/absence as input (Fig. 1). Both the PCA and the hierarchical clustering show a separation between several facultative anaerobic strains and the rest of the considered strains (among which *P. putida* KT2440). However, it should be noted that only a small fraction of the total variance is explained by the first two principal components. This separation is also apparent in the dendrogram, suggesting that significant differences could be found in protein domain content.

**Fig. 2:**
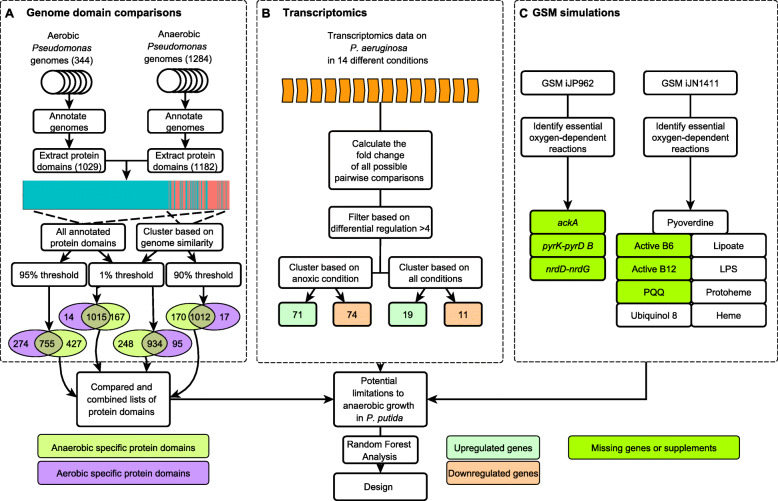
Overview of in silico approaches to identify limitations to anaerobic respiration in P. putida. **a** Comparative genomics workflow. Genomes of the P. putida group and the anaerobic Pseudomonas group were systematically annotated using SAPP [24, 29], the protein domains were extracted, and both all domains or only the domains common to all anaerobic Pseudomonas species (the core domains) were selected using a 95% persistence threshold. Analysis was performed on the whole set of genomes (left) or a genome cluster of closely related strains (right). Each of these methods resulted in a list of protein domains related to an aerobic lifestyle (purple) or an anaerobic lifestyle (light green). **b** Transcriptome analysis. **c** GSM simulations. GSM iJP962 [5] and iJN1411 [35] were expanded with indicated reaction sets and tested for anaerobic growth under anaerobic conditions. Colours indicate final implementation in the design (green). Model and genome base predictions were combined to obtain a final design

We assumed that domains essential for anaerobic respiration are highly persistent in facultative anaerobic strains but show a lower persistence in obligate aerobic strains. The strategy to obtain this protein domain core is outlined in Fig. 2. A “long list” of anaerobic protein domains was generated by comparing domain persistence between aerobic versus anaerobic strains. First a 95% persistence threshold was applied, to obtain a “domain core” of domains present in at least 95% of the genomes of “aerobic” strains and in the “anaerobic” strains analysed. These aerobic and anaerobic domain cores were used as input for subsequent comparative analysis and for the first list were split into “shared between aerobic and anaerobic species” (Shared domain core), “specific for aerobic species” (Aerobe specific domain core) and “specific for anaerobic species” (Anaerobe specific domain core) creating a long list of 427 anaerobe specific protein domains. A second long list was created by the same input but searching for the reverse, a separation based on domains with a very low persistency in aerobic or anaerobic strains. For this a no more than 1% threshold was applied creating a long list of 167 anaerobe specific protein domains.

The dendrogram presented in Fig. 1 indicated a possible early branch split between a large group of exclusively anaerobic *Pseudomonas* strains and a mixed group, including *P. putida* KT2440, containing 138 facultative anaerobic and 87 obligatory aerobic *Pseudomonas* strains (Fig. 1 panel C). Using this split, two “restricted” lists were built by comparing domain persistence as outlined above, but now evaluating only *Pseudomonas* strains present in the mixed branch. For the restricted lists, a 90% persistence threshold and a 1% persistence threshold were used, creating two anaerobic species-specific protein domains lists of 170 and 248 domains, respectively. The four different lists of protein domains essential for anaerobic growth were compared and manually further annotated. Results are summarized in Table 1 and Fig. 2.

**Table 1:** Respiratory design of a facultative anaerobic P. putida KT2440. Number of unique protein domains obtained

Method	# Unique protein domains
**Genome domain comparisons**	
Input aerobic domain core	1029
Input anaerobic domain core	1182
**Long list, 1628 strains [95% threshold]**	
Shared domain core	755
Aerobic specific domain core	274
Anaerobic specific domain core	427
**Long list, 1628 strains [1% threshold]**	
Shared domain core	1015
Aerobic specific domain core	14
Anaerobic specific domain core	167
**Restricted list, 225 strains [90% threshold]**	
Shared domain core	1012
Aerobic specific domain core	17
Anaerobic specific domain core	170
**Restricted list, 225 strains [1% threshold]**	
Shared domain core	934
Aerobic specific domain core	95
Anaerobic specific domain core	248
**Transcriptome analysis**	175
**GSM simulations**	18
**RandomForest [input]**	5831
Domains with a cumulative Gini coefficient ≥ 20	360
Domains with a cumulative Gini coefficient ≥ 100	5

As outlined in the Materials and Methods section, the domain content of the facultative anaerobic and the obligatory aerobic *Pseudomonas* strains were used to train a random forest classifier with the goal to identify those domains (features) that are mostly responsible for classification. Gini coefficients and cumulative Gini coefficients for each domain are provided in Data S9. From the 5831 domains that were used as input for the classifier, 5 have a cumulative Gini coefficient ≥ 100, as summarized in Table 1. Gini scores were added as weight to the four protein domain lists derived above.

Transcriptome data obtained from *P. aeruginosa* PO14 grown under 14 different environmental conditions including anoxic conditions [25] was re-analysed for genes that were consistently differentially expressed during anaerobic respiration (see the Materials and Methods section for details). By calculating for every gene, the log2fold change of its expression values in every possible condition compared with anaerobic respiration, 175 protein domains were identified. A heatmap was used to visualise up- and down-regulated genes under anoxic conditions. Regulation due to anoxic growth was considered to be significant when the same behaviour (up- or down-regulation) was observed in at least 7 of the 13 pair-wise comparisons and a fold change of at least 4 was observed in at least three of these comparisons. Protein domain architectures corresponding to the selected locus tags were identified. Based on the differential expression and similar efforts in literature [13], 22 genes encompassing 35 protein domains were selected.

Genome-scale models were used to simulate anoxic conditions. The absence of any reaction products impeding growth due to the simulated lack of oxygen were pinpointed and traced back to proteins and their encoding genes that either need oxygen as a substrate or that cannot be made without oxygen present, resulting in substrates that could thus not be produced under anoxic conditions. Genes and substrates were verified through literature analysis to be essential for growth (Table 1).

### Design considerations

A comparison was made between the different lists obtained (Table 1) and previous efforts [4, 13, 14, 23] resulting in an extensive overview of the many hurdles to overcome to build a *P. putida* KT2440 strain capable of anaerobic respiration. The various lists were compared by evaluating the function of each gene starting with the encoded domain annotation, checking for domain co-existence in operonic structures, comparing metabolic functions with GSM data and with gene regulation data. The importance of each protein domain was determined using the random forest analysis (Data S9) as input. In this way, different lists could be combined and reduced to a list of 57 genes. Furthermore, a supplement of 3 vitamins is required.

The selected genes can be separated into various categories based on their functions: Nitrogen metabolism (45 domains in 35 genes), Hydrogenases (9 domains in 9 genes), Cytochrome C (1 domains in 1 genes), Pyrimidine and amino acid biosynthesis (6 domains in 3 genes if 3 vitamins added), ATP production (1 domains in 1 genes), and Domains of Unknown Function (indirectly associated with anaerobic respiration) (8 domains).

#### Nitrogen metabolism

Most *Pseudomonas* species capable of anaerobic respiration do so using nitrite or nitrate as alternative terminal electron acceptor. Of the 49 known genes found vital for anaerobic respiration, 35 are either directly or indirectly involved in nitrogen metabolism. With nitrate as the final electron acceptor in anaerobic respiration the largest amount of energy can be conserved when compared to other final electron acceptors such as sulphate, iron (III), manganese (II), or selenate [37]. *P. putida* KT2440 lacks the nitrate/nitrite respiration pathway, which was resolved in earlier studies by inserting either a Nir-Nar or a Nor plasmid [13]. This resulted in extended survival under anoxic conditions, but not to growth. Our transcriptomics and protein domain analysis indicated that the combination of the operons of both the Nir-Nar and the Nor operon are required (Table 2). The operons include genes of the Nif, RhfH, Nqr, Rnf, Dau, Nar, Nir and Nor, and Moa protein families, and are required for energy conservation, cofactor biosynthesis, amino acid biosynthesis, nitrogen metabolism, nitrate-, nitrite- and nitrogen transporters, nitrate-, nitrite-, nitric oxide and nitrous oxide reductases and several regulatory proteins (Table 2 and Additional file 18). Of the 45 protein domains or 35 genes we identified within this category, only 15 genes had been previously found (*narK1, narK2, narG, narH, narJ, narI narX, narL, nirF, nirQ, nirM, nirS, nirJ, nirL* within the Nir-Nar operon, *norC, norB, norD, nosR* within the Nor operon) [13]. Ureohydrolases such as Arg1, SpeB, HutG and Pah facilitate the ammonia to urea conversion, with urea as the principle product of nitrogen excretion.

**Table 2:** Respiratory design of a facultative anaerobic P. putida KT2440. Genes related to nitrogen metabolism

InterPro Entry	PFAM	Name	Abbreviation	Function	Source
	PF00491	**ureohydrolases**		Ammonia to urea conversion	PDC
		Arginase	ArgI	L-arginine + H(2) O < => L-ornithine + urea	PDC
		Agmatinase	SpeB	Agmatine + H(2) O < => putrescine + urea	PDC
		Formimidoylglutaminase	HutG	N-formimidoyl-L-glutamate + H(2) O < => L-glutamate + formamide	PDC
IPR015868	PF04960	Glutaminase		Glutamine + H2O → Glutamate + NH3	PDC, T
IPR000292	PF01226	Formate/nitrite transporter		Transporter	PDC, T
IPR025736	PF13556	PucR C-terminal helix-turn-helix domain	PucR	PucR-like transcriptional regulator	PDC, T
IPR000825	PF01458	**Uncharacterized protein family (UPF0051)**		Chaperone proteins for nitrogenase production	PDC, T
			NifS	Metallocluster formation Nitrogenase	PDC, T
			NifU	Metallocluster formation Nitrogenase	PDC, T
IPR005346	PF03658	Ubiquitin	RnfH family	Electron transport	PDC, T
	PF02508	Rnf-Nqr subunit, membrane protein	Rnf-Nqr	Nitrogen fixation	PDC, T
	PF03116	**Nqr2 family**	Nqr2	Nitrogen fixation	PDC, T
		RnfD family	RnfD	Nitrogen fixation	PDC, T
		RnfE family	RnfE	Nitrogen fixation	PDC, T
	PF03060	Nitronate monooxygenase		Nitrogen metabolism	PDC, T
IPR010349	PF06089	L-asparaginase II		Hydrolysis of L-asparagine to L-aspartate and ammonium.	PDC, T
PA3862	PF02423		DauB	NAD(P)H-dependent anabolic L-arginine dehydrogenase	PDC, T, Steen et al., 2012
PA3863	PF01266		DauA	FAD-dependent catabolic D-arginine dehydrogenase	PDC, T, Steen et al., 2012
PA3864	PF08348		DauR	Transcriptional regulator of the dauBAR operon	PDC, T, Steen et al., 2012
	PF13309				PDC, T, Steen et al., 2012
PA14_13750	PF07690	Nitrite extrusion protein (putative)	NarK2	Membrane protein; Transport of small molecules	PDC, T, Steen et al., 2012
PA14_13770	PF07690	Nitrite extrusion protein	NarK1	Membrane protein; Transport of small molecules	PDC, T, Steen et al., 2012
PA3875	PF14710		NarG	Energy metabolism	PDC, T, Steen et al., 2012
	PF00384				PDC, T, Steen et al., 2012
	PF01568				PDC, T, Steen et al., 2012
PA14_13800	PF13247	Nitrate reductase	NarH	β-subunit, Energy metabolism	PDC, T, Steen et al., 2012
	PF14711				PDC, T, Steen et al., 2012
PA14_13810	PF02613	Nitrate reductase	NarJ	Δ-chain, Energy metabolism	PDC, T, Steen et al., 2012
PA14_13830	PF02665	Nitrate reductase	NarI	γ-chain, Energy metabolism	PDC, T, Steen et al., 2012
PA3878	PF02518	two-component sensor	NarX		PDC, T, Steen et al., 2012
	PF00672				PDC, T, Steen et al., 2012
	PF07730				PDC, T, Steen et al., 2012
	PF13675				PDC, T, Steen et al., 2012
PA3879	PF00072	two-component response regulator	NarL	Response regulator	PDC, T, Steen et al., 2012
	PF00196				PDC, T, Steen et al., 2012
PA14_13850	PF04055	Heme d1 biosynthesis protein	NirJ	Heme d1 Biosynthesis	PDC, T, Steen et al., 2012
PA0516	PF02239	Heme d1 biosynthesis protein	NirF	Heme d1 Biosynthesis	PDC, T, Steen et al., 2012
PA0514		Heme d1 biosynthesis protein	NirL	Heme d1 Biosynthesis	PDC, T, Steen et al., 2012
PA0520	PF07728		NirQ	Regulatory protein	PDC, T, Steen et al., 2012
	PF08406		CbbQ	post-translational activation of Rubisco -- photosynthesis	PDC, T, Steen et al., 2012
			NorQ,	Post-translational activation of Rubisco -- photosynthesis	
PA14_06750	PF13442	nitrite reductase precursor	NirM	Cofactor biosynthesis, Energy metabolism	PDC, T, Steen et al., 2012
PA3870	PF00994	molybdopterin biosynthetic protein A1	MoaA1	Biosynthesis of cofactors, prosthetic groups and carriers	PDC, Steen et al. 2012
	PF03453				PDC, Steen et al. 2012
	PF03454				PDC, Steen et al. 2012
PA14_13260	PF00994	molybdopterin biosynthetic protein B1	MoaB1		PDC
	PF00394	**Multicopper oxidase**			PDC, T
PA0519	PF13442	Nitrate reductase	NirS	Energy metabolism	PDC, T, Steen et al., 2012
		Nitrate reductase	NirS	Energy metabolism	PDC, T, Steen et al., 2012
	PF02239	Nitrate reductase	NirS	Energy metabolism	PDC, T, Steen et al., 2012
	PF05940	**NnrS protein**			PDC, T
			NirK	Reduction of nitrite to nitrous oxide	PDC, T
			Nor	Reduction of nitrite to nitrous oxide	PDC, T
PA14_06810	PF00034	nitric-oxide reductase	NorB-NorC	Subunit B, C	PDC, T, Steen et al., 2012
PA14_06830	PF00115	nitric-oxide reductase	NorB-NorC	Subunit B, C	PDC, T, Steen et al., 2012
PA14_06840	PF00092		NorD	Putative dinitrification protein	PDC, T, Steen et al., 2012
PA14_20230	PF04205		NosR	Regulatory protein for N2O reductase	PDC, T

*T* Trancriptomics, *PDC* Protein Domain Comparisons, *GSM* Genome Scale Modelling [30, 31];. Printed in bold are classes of genes, the genes belonging to that class listed directly underneath

#### Hydrogenases

Included in the anaerobic respiration design are 9 hydrogenase subunits: HupH, HypA-F, HyaE, and HybE. Hydrogenases catalyse the reversible oxidation of molecular hydrogen, fulfilling a regulatory role in balancing the redox state. The redox state of the cell and the availability of O2 are regulatory signals in facultative anaerobic species [38]. [FeFe] And [NiFe]-hydrogenases are widely distributed under anaerobic species. These hydrogenases are only produced under anoxic conditions, and most [NiFe]-hydrogenases are inactivated by oxygen, only to be re-activated under reducing conditions [39].

*P. putida* KT2440 lack hydrogenases necessary for proton reduction or coupling H_2_ oxidation to energy yielding processes under anoxic conditions, and the necessary hydrogenase chaperones, assembly, maturation and formation proteins (Table 3).

**Table 3:** Respiratory design of a facultative anaerobic P. putida KT2440. Genes encoding for hydrogenases

InterPro Entry	PFAM	Name	Abbreviation	Function	Source
IPR027394	PF14720	NiFe/NiFeSe hydrogenase		Reversible oxidation of molecular hydrogen	PDC
	PF09459	Ethylbenzene dehydrogenase		Anaerobic degradation of hydrocarbons	PDC
IPR001501	PF00374	Nickel-dependent hydrogenase		Catalysis the reversible activation of hydrogen	PDC
IPR001109	PF01455	HupF/HypC family	HupF/HypC	[NiFe]-hydrogenase and other nickel metalloenzymes synthesis	PDC
IPR000671	PF01750	Hydrogenase maturation protease		Hydrogenase maturation	PDC
IPR002780	PF01924	Hydrogenase formation	HypA	Hydrogenase formation	PDC
IPR006894	PF04809	Hydrogenase expression protein	HupH	Hydrogenase synthesis, C-terminal domain	PDC
IPR010893	PF07449	Hydrogenase-1 expression protein	HyaE	Hydrogenase assembly	PDC
IPR023994	PF11939	Chaperone for [NiFe]-hydrogenase assembly	HybE	[NiFe] hydrogenases assembly chaperone	PDC
	PF13237	4Fe-4S dicluster domain		Mediate electron transfer	PDC
IPR000688	PF01155	Metallochaperone	hypA	[Ni,Fe]-hydrogenase and urease chaperone	PDC
IPR007038	PF04955	HupE / UreJ protein	HupE / UreJ	Hydrogenase / urease accessory proteins	PDC

*T* Trancriptomics, *PDC* Protein Domain Comparisons, *GSM* Genome Scale Modelling [30, 31];

None of these genes have been recognised in previous research for their importance in anaerobic respiration.

#### Cytochrome C

Included in the anaerobic respiration design are 3 C-type cytochromes. C-type cytochromes account for a vital step in ATP bio-generation via the proton motive force (Table 4). Anaerobically, cytochrome C 551 (NirN), C 552 transfer electrons to nitrite reductase (NirS) and nitric-oxide reductase (NorB, NorC). The importance of NirN and NirC (the precursor of NirN) was demonstrated in [13] (Table 2).

**Table 4:** Respiratory design of a facultative anaerobic P. putida KT2440. Genes related to cytochrome C

InterPro Entry	PFAM	Name	Abbreviation	Function	Source
IPR003321	PF02335	Cytochrome c552		Cytochrome C oxidase bio generation	PDC

*T* Trancriptomics, *PDC* Protein Domain Comparisons, *GSM* Genome Scale Modelling [30, 31];

The PDC also indicates the need for cytochrome C 552 (Tables 2, 4). The enzyme cytochrome C nitrite reductase (C 552), amongst other important functions, catalyses the six-electron reduction of nitrite to nitrogen as one of the key steps in denitrification. Nitrogen is then reduced to ammonium in the ammonification pathway. C552 thus participates in the anaerobic energy metabolism of dissimilatory nitrate ammonification.

In addition, cobalamin-independent methionine synthase is important. This methionine synthase is responsible for precursor formation of C 551 that can be produced without using vitamin B12 (see Pyrimidine and amino acid biosynthesis, Table 5). This might be a key component for anaerobic growth, since both the protein domain analysis and the GSM iJN1411 [35] predict that, amongst other vitamins, the active form of vitamin B12 can only be bio-generated in the presence of oxygen in *P. putida* KT2440.

**Table 5:** Respiratory design of a facultative anaerobic P. putida KT2440. Genes related to pyrimidine and amino acid biosynthesis

InterPro Entry	PFAM	Name	Abbreviation	Function	Source
IPR002751	PF01891	Cobalt uptake substrate-specific transmembrane region	Vitamin B12 ^a^	VitB12 Biosynthesis	PDC
IPR006538	PF06213	Cobalamin biosynthesis protein CobT	Vitamin B12 ^a^	VitB12 Biosynthesis	PDC
	PF09489	Probable cobalt transporter subunit (CbtB)	Vitamin B12 ^a^	VitB12 Biosynthesis	PDC
	PF10531	SLBB domain	Vitamin B12 ^a^	Vit B12 uptake	PDC
		adenosylcobalamin	Vitamin B12 ^a^	VitB12 Biosynthesis	GSM
	PF02621	Menaquinone biosynthesis	Vitamin K2 ^a^	VitK2 biosynthesis	PDC
		Pyridoxal-5-phosphate	Vitamin B6 ^a^	Pyridoxal-5-phosphate biosynthesis	GSM
		Dihydroorotate dehydrogenase	PyrK-PyrD B	Pyrimidine production	GSM, [23]
		Ribonucleotide triphosphate reductase type II	NrdD-NrdG	Pyrimidine production	GSM, [23]
PA0527	PF00027	transcriptional regulator	DNR	Transcriptional regulators	Steen et al. 2012
	PF13545				Steen et al. 2012

*T* Trancriptomics, *PDC* Protein Domain Comparisons, *GSM* Genome Scale Modelling; ^a^can be added as vitamin to medium [30, 31];

#### Pyrimidine and amino acid biosynthesis

Included in the anaerobic respiration design are 2 genes involved in pyrimidine and amino acid synthesis, and additional bottlenecks that can be solved by adding 3 vitamins to the medium. Earlier GSM simulations with iJP962 indicated that alternate genes must be inserted for dihydroorotate dehydrogenase and ribonucleotide triphosphate reductase type II for pyrimidine and ultimately DNA and RNA biosynthesis [23]. Both the protein domain analysis and GSM simulations using the iJN1411 metabolic model predicted that cobalamin (vitamin B12), pyridoxal-5-phosphate (vitamin B6) and menaquinone (vitamin K2) cannot be produced under anoxic conditions.

Crespo et al. showed that class II RNRs depend on adenosylcobalamin or vitamin B12 (cobalamin) to generate its radical independently of oxygen [40]. Cobalamin is a complex essential cofactor for many enzymes mediating methylation, reduction, and intramolecular rearrangements, and for methionine synthase. There is a recognised distinction between aerobic and anaerobic generation of cobalamin [41, 42]. The routes differ in terms of cobalt chelation (via CobNST complex in the aerobic pathway, via precorrin-2 with CbiK in the anaerobic pathway) and oxygen requirements. The enzymes CobI, CobG, CobJ, CobM, CobF, CobK, CobL, CobH, CobB and CobNST form the aerobic pathway. CbiK, CbiL, CbiH, CbiF, CbiG, CbiD, CbiJ, CbiET, CbiC and CbiA form the anaerobic route [31, 41, 43]. Surprisingly, the protein domain comparison yielded none of the enzymes of the anaerobic pathway for vitamin B12 synthesis, but instead CobT and CbtB, both described as important for the aerobic pathway [41]. According to the extensive analysis, these specific protein domains linked to these genes are not present in aerobic species analysed but only in anaerobic species. It was found that in the anaerobic bacterium *Eubacterium limosum*, CobT functions as an activator for a range of lower ligand substrates including DMB, determining cobamide diversity. The specific function of CbtB is unknown [41, 42].

Vitamin B6 is required for a wide variety of processes [44]. There are many vitamin B6-dependent proteins involved in amino acid biosynthesis, amino acid catabolism, antibacterial functions, iron metabolism, carbon metabolism, nucleotide utilization, cofactors for biotin, folate and heme, NAD biosynthesis, cell wall metabolism, tRNA modification, regulation of gene expression and biofilm formation.

Vitamin K2 is responsible for electron transport during anaerobic respiration. However, knock-out experiments in *E. coli* showed that upon loss of menaquinone and vitamin K1 only 3% of theoretical yield was obtained, but this was instantly revived to 44% upon supplementing of vitamin K1 or vitamin K2 [45], indicating vitamin K1 can partially make up for the loss of vitamin K2.

Rather than inserting all missing genes, in a minimal design setup, these vitamins can be supplemented to the medium (indicated in Table 5 with ∗). To determine any immediate effect on growth or survival rates, medium supplementation of these vitamins was tested, monitoring performance of all recombinant strains under anoxic conditions. In parallel a survival experiment without the vitamin mix was done. No difference in growth or survival rates was found (Figure S4, Figure S5, Data S10, Data S11).

Lastly within this category, transcriptional regulator DNR was found, its importance in anaerobic respiration described in earlier research [13].

#### ATP generation

Of the 49 genes of known function required for anaerobic respiration, only one is involved in ATP generation. The protein domain analysis, transcriptomics data and metabolic modelling with iJP962 and iJN1411 all indicated that ATP production remains one of the main bottlenecks to tackle. Earlier work came to the same conclusion and this was tackled by insertion of genes for acetate production [4, 14]. The recombinant strain best performing in previous micro-oxic research included acetate kinase (*ackA)* for ATP generation through acetate production, which was therefore included in the previous design [23] (Table 6). *Pseudomonas putida* KT2440 has its own functional phosphate acetyltransferase (*pta*).

**Table 6:** Respiratory design of facultative anaerobic P. putida KT2440. Genes related to ATP generation to add for a P. putida KT2440 capable of anaerobic respiration

InterPro Entry	PFAM	Name	Abbreviation	Function
	Acetate kinase	AckA	ADP to ATP conversion by acetate production	PDC, GSM, Kampers et al. under review

*T* Trancriptomics, *PDC* Protein Domain Comparisons, *GSM* Genome Scale Modelling [30, 31];

#### Domains of unknown function

The protein domain analysis additionally included 270 unique protein domains of unknown function occurring in the genomes of facultative anaerobic strains but not in aerobic strains. Based on contextual information, eight were identified as important for anaerobic respiration by their physical co-localisation with genes required for anaerobic respiration. These were therefore included in the design (Table 7). Similarly, 28 protein domains of unknown function were associated with virology factors or immunity and on these ground were excluded from the design. The remaining 234 protein domains of unknown function provided no direct contextual hints and thus cannot not be conclusively excluded from the design.

**Table 7:** Respiratory design of facultative anaerobic P. putida KT2440. Domains of unknown function to add for a P. putida KT2440 capable of anaerobic respiration

InterPro Entry	PFAM	Name	Abbreviation	Function	Source
	PF09086	Domain of unknown function	DUF1924		PDC
IPR013039	PF07627	Domain of unknown function	DUF1588		PDC
IPR013036	PF07626	Domain of unknown function	DUF1587		PDC
IPR013042	PF07631	Domain of unknown function	DUF1592		PDC
IPR013043	PF07637	Domain of unknown function	DUF1595		PDC
IPR011727	PF09601	Domain of unknown function	DUF2459		PDC
	PF12981	Domain of unknown function	DUF3865		PDC
	PF02026	Domain of unknown function	RyR domain		PDC

*T* Trancriptomics, *PDC* Protein Domain Comparisons, *GSM* Genome Scale Modelling [30, 31];

## Discussion

### No extended survival under anoxic conditions after acetate kinase expression

Our previous rational design [23] was based on two genome-scale models and genome domain comparison analysis of six facultative anaerobic *Pseudomonas* species compared to six obligatory aerobic *Pseudomonas putida* species. Under micro-oxic conditions, the addition of acetate kinase, dihydroorotate dehydrogenase and class II ribonucleotide triphosphate reductase leads to growth.

In our hands there was however no extended survival under anoxic conditions of the recombinant strains upon introduction of *ackA*. It is extremely challenging to acquire anoxic conditions. Both the medium and the headspace must be treated to completely remove oxygen from the start of the experiment, otherwise oxygen depletion takes up to 12 h. Further, the medium must be prepared with L-cysteine or sodium thioglycollate to actively remove oxygen. Without these precautions, the medium is very easily oxygenated. Small stopper-capped vials are preferred strongly over screw-cap vials, in which oxygen leaks frequently occurred [23]. Addition of the redox-sensitive phenoxazine dye resazurin functions to detect aerobic respiration. Resazurin is generally used as an oxygen indicator as the colour changes from dark purple (high oxygen levels) to pink (low oxygen levels) to transparent (below detectable oxygen levels, as determined by micro-electrode at 0.01 g/l dissolved oxygen [23]). However, resazurin cannot be applied to distinguish between micro-oxic and anoxic conditions.

The lack of improvement in survival rates under anoxic conditions can easily be explained when contemplating the novel design assembled in this research, as numerous essential factors such as an alternative electron acceptor or an anaerobically active cytochrome-C were missing.

### Technical design issues

The aim of this research was to determine the requirements for anaerobic respiration, using the industrially interesting workhorse *P. putida* KT2440 as a concept organism. This fundamental question resulted in a design that required 49 genes of known function and 8 genes encoding protein domains of unknown function, resulting in almost 60 genes to be included in the genome. For solely industrial applications, it might be more beneficial to opt for a facultative anaerobic strain from the start. However, this design does offer a fundamental insight in the elaborate change in genotype to achieve an anaerobic lifestyle.

To enable an anaerobic lifestyle, previous designs included the introduction of between 3 and 24 genes in *P. putida* KT2440 genome [4, 13, 14, 23] but our in silico methods suggests that approximately three times more genes are required. Novel methods developed specifically for integration of large operons or multiple genes like yTREX [46] allow incorporation of up to 14 genes at the time in *P. putida*. Albeit an elaborate effort, we consider the inclusion of almost 60 genes in the *P. putida* KT2440 genome to be technically possible, especially since there are constantly new developments in the field of genome engineering.

The GSMs indicated that to produce vitamin B6 and vitamin K2, oxygen is indirectly used. Although anaerobic alternative routes could be implemented, supplementation of these vitamins in the medium significantly reduces the design. Furthermore, while the design is based on gene presence or absence in anaerobic strains of the same species, the design could be slimmed down by a futher elimination of proposed genes based on more elaborate analyses of their gene activity in facultative anaerobic species when compared to aerobic species. This would for instance include a selection of nitrogen sources the strain would grow on and would serve to eliminate all nitrogen fixation genes up to that point.

The 57 genes in our design do not consider the 234 genes of unknown function, which complicate the task even further. Without knowing their function, these genes cannot be excluded from the design. At least eight of these were found to be closely associated with genes required for growth in anoxic conditions in literature and/or through their physical co-localisation [31]. The crucial roles that genes of unknown function might play was demonstrated by Hutchison and colleagues [47], who in their attempt to make a minimal bacterial genome, unexpectedly found 149 genes of unknown function to be essential for growth.

Many of the genes found in the design are associated with metal chelation and transport, including many hydrogenases and genes required for vitamin biosynthesis. It should be considered that changes in oxygen availability drastically alters metal bioavailability as extensively reviewed in [48]. Interestingly, we found multiple strict aerobic *Pseudomonas* strains which upon literature research proved able of nitrogen fixation, but not of anaerobic respiration. This might suggest that the evolutionary road between the two first includes nitrogen fixation (or excludes ammonification) before including ammonification (or excluding nitrogen fixation). Either way, experimental validation in anaerobic strains is required to proof which genes required for ammonification or and nitrogen fixation are essential for anaerobic respiration.

### The new design compared to previous designs

We predicted that for anaerobic growth both the Nir-Nar and Nor operons are vital. There do exist *Pseudomonas* species that naturally have only one of these operons and are capable of nitrate to nitrite transformation. However, these strains respire nitrate under oxic conditions only, and have been shown to be incapable of growth in anoxic conditions [49, 50]. If *P. putida* KT2440 would be enriched with both the denitrification pathway and the ammonification pathway it could reduce nitrate or nitrite to ammonium, which could then be assimilated to organic compounds, transforming *P. putida* KT2440 in a diazotroph of agronomic importance [51].

The most prevalent anaerobic dissimilatory nitrate respiration regulator DNR is a key transcription factor obtained from the protein domain comparison. In the facultative anaerobic *E. coli*, knock-out FNR mutants, an ortholog of DNR, were unable to grow by anaerobic respiration under anoxic conditions. By DNA microarray technology it was shown that in *E. coli* 49% of the genes that differ in expression between anoxic and oxic conditions are regulated by FNR [38]. The two-component aerobic respiratory control system (ArcA and ArcB) controls gene transcription in *E. coli* under anoxic conditions. Mutations in this system are known to affect expression of over 30 operons. Most of these are repressed under anoxic conditions, but cytochrome C oxidase and pyruvate formate lyase are activated. In *E. coli*, ArcAB and FNR are deemed essential for anaerobic activation and robustness under micro-oxic conditions [52–55]. To maintain after incorporating the ability of anaerobic respiration, optimal functionality of this strain under oxic conditions, these genes are included in the final design.

We argue that for a lifestyle shift from a strict aerobic lifestyle in *P. putida* KT2440 to an faculatitve anaerobic respiratory lifestyle, all 49 known genes, at least 8 protein domains of unknown function and 3 added vitamins are required. However, increased strain performance under micro-oxic conditions or prolonged survival rates under anoxic conditions already could significantly improve strain robustness in large scale bioreactors with fluctuating oxygen levels. For enhanced performance under micro-oxic conditions, it was demonstrated that increasing ATP production through acetate production already appears to be enough [23]. For prolonged survival rates, however, these key elements include both Nir-Nar and Nor operons for denitrification and ammonification, cytochrome C 552, and external supplementation of the lacking vitamins. This conclusion is supported by previous findings that energy supply and redox balancing are the main bottlenecks in an anaerobic lifestyle [4, 13–16, 23].

## Conclusion

Increased ATP generation by insertion of acetate kinase via a plasmid does not lead to prolonged survival rates of *Pseudomonas putida* KT2440 under anoxic conditions. This proves that increased performance under micro-oxic conditions does not guarantee prolonged survival under anoxic conditions. A *P. putida* KT2440 strain capable of anaerobic respiration would require the insertion of at least 57 additional genes into the genome and a supplement of 3 vitamins to the medium. The conversion of a strict aerobic species to a facultative anaerobic lifestyle by anaerobic respiration is a much more elaborate process than was thought before. Especially the function of DUFs and their role in anaerobic respiration must be researched, as it remains unknown how many of these should be added to this design.

## Supplementary Information

**Additional file 1: Table S1.** Bacterial strains and plasmids used in this study .xlsx file with bacterial strains and plasmids listed, including references or sources

**Additional file 2: Figure S1.** Anoxic survival of P. putida KT2440 transformed strains, grown on De Bont minimal medium with gluconic acid as sole carbon source and kanamycin. The headspace was flushed from oxygen with nitrogen. Survival under anoxic conditions was determined by comparing the number of colony forming units (CFU) over time with the number of CFU at T0. *Escherichia coli* BW25113 was used as positive control and Pseudomonas putida KT2440 with an empty plasmid (pS2213 -) was used as a negative control. Tested strains were Pseudomonas putida KT2440 with acetate kinase (pS2213 ackA) unpassed (p + 0) or passed three consecutive times over oxygen gradients (p + 3), and Pseudomonas putida KT2440 with acetate kinase, dihydroororotate dehydrogenase and ribonucleotide triphosphate reductase type II (pS2213 ackA-(pyrK-pyrD B)-(nrdD-nrdG) unpassed (p + 0) or passed three consecutive times over oxygen gradients (p + 3).

**Additional file 3: Figure S2.** Survival experiment of P. putida KT2440 under anoxic conditions. The CFU determination of Pseudomonas putida KT2440 with an empty plasmid (pS2213 -), acetate kinase (pS2213 ackA) or acetate kinase, dihydroororotate dehydrogenase and ribonucleotide triphosphate reductase type II (pS2213 ackA-(pyrK-pyrD B)-(nrdD-nrdG) unpassed (p + 0) or passed three consecutive times over oxygen gradients (p + 3) survival under anoxic conditions. The experiment was repeated independently six times. All figures share the same legend. (A) Experiment 1 (B) Experiment 2 (C) Experiment 3 (D) Experiment 4.

**Additional file 4: Figure S3.** Transcriptomics of *Pseudomonas aeruginosa* PA01 in 15 different conditions. (A) Heatmap of up (green) or downregulation (red) of all genes per condition. (B) All upregulated genes per condition. (C) All downregulated genes per condition.

**Additional file 5: Figure S4.** Survival experiment of P. putida KT2440 under anoxic conditions. The CFU determination of Pseudomonas putida KT2440 with an empty plasmid (pS2213 -), acetate kinase (pS2213 ackA) or acetate kinase, dihydroororotate dehydrogenase and ribonucleotide triphosphate reductase type II (pS2213 ackA-(pyrK-pyrD B)-(nrdD-nrdG) unpassed (p + 0) or passed three consecutive times over oxygen gradients (p + 3) with or without vitamin mix.

**Additional file 6: Figure S5.** Growth experiment of P. putida KT2440 under anoxic conditions. The OD600 determination of Pseudomonas putida KT2440 with an empty plasmid (pS2213 -), acetate kinase (pS2213 ackA) or acetate kinase, dihydroororotate dehydrogenase and ribonucleotide triphosphate reductase type II (pS2213 ackA-(pyrK-pyrD B)-(nrdD-nrdG) unpassed (p + 0) or passed three consecutive times over oxygen gradients (p + 3) with vitamin mix.

**Additional file 7: Data S1.** With anaerobic cultivation Analysis 1.

**Additional file 8: Data S2.** With anaerobic cultivation Analysis 2.

**Additional file 9: Data S3.** With anaerobic cultivation Analysis 3.

**Additional file 10: Data S4.** With anaerobic cultivation Analysis 4.

**Additional file 11: Data S5.** With *Pseudomonas* selection database as obtained via GOLDDatabase, including extra information and sources.

**Additional file 12: Data S6.** With PDC data.

**Additional file 13: Data S7.** With Transcriptomics data.

**Additional file 14: Data S8.** With GSMsimulation data.

**Additional file 15: Data S9.** With random forest data.

**Additional file 16: DataS10.** With anaerobic cultivation Analysis 5.

**Additional file 17: DataS11.** With anaerobic cultivation Analysis 6.

**Additional file 18.** Potential roles of Dau, Nif, Rnf, RhfH operons in anaerobic respiration.

## Data Availability

All data generated or analysed during this study is included in this published article and its supplementary information files.

## References

[1] Kampers LFC, Volkers RJM, Martins dos Santos VAP (2019). *Pseudomonas putida* kt2440 is hv1 certified, not gras. Microb Biotechnol.

[2] Belda E, van Heck RGA, Jos’e Lopez-Sanchez M, Cruveiller S, Barbe V, Fraser C (2016). The revisited genome of *Pseudomonas putida* KT2440 enlightens its value as a robust metabolic chassis. Environ Microbiol.

[3] Nogales J, Palsson BO, Thiele I (2008). A genome-scale metabolic reconstruction of *Pseudomonas putida* KT2440: i JN746 as a cell factory. BMC Syst Biol.

[4] Sohn SB, Kim TY, Park JM, Lee SY (2010). *In silico* genome-scale metabolic analysis of *Pseudomonas putida* KT2440 for polyhydroxyalkanoate synthesis, degradation of aromatics and anaerobic survival. Biotechnol J.

[5] Oberhardt MA, Puchalka J, Martins dos Santos VAP, Papin JA (2011). Reconciliation of genome-scale metabolic reconstructions for comparative systems analysis. PLoS Comput Biol.

[6] Puchalka J, Oberhardt MA, Godinho M, Bielecka A, Regenhardt D, Timmis KN (2008). Genome-scale reconstruction and analysis of the *Pseudomonas putida* KT2440 metabolic network facilitates applications in biotechnology. PLoS Comput Biol.

[7] van Heck RG, Ganter M, dos Santos VAM, Stelling J (2016). Efficient reconstruction of predictive consensus metabolic network models. PLoS Comput Biol.

[8] Clarke PH (1982). The metabolic versatility of pseudomonads. Antonie Van Leeuwenhoek.

[9] Bagdasarian M, Lurz R, Ruckert B, Franklin FCH, Bagdasarian MM, Frey J (1981). Specific-purpose plasmid cloning vectors II. Broad host range, high copy number, RSF 1010-derived vectors, and a host-vector system for gene cloning in *Pseudomonas*. Gene.

[10] Nikel PI, de Lorenzo V (2018). *Pseudomonas putida* as a functional chassis for industrial biocatalysis: from native biochemistry to trans-metabolism. Metab Eng.

[11] Avendano R, Chaves N, Fuentes P, S’anchez E, Jim’enez JI, Chavarr’ıa M (2016). Production of selenium nanoparticles in *Pseudomonas putida* kt2440. Sci Rep.

[12] Poblete-Castro I, Becker J, Dohnt K, Santos VM, Wittmann C (2012). Industrial biotechnology of *Pseudomonas putida* and related species. Appl Microbiol Biotechnol.

[13] Steen A, Utkur FO, Borrero-de Acuna JM, Bunk B, Roselius L, Buhler B (2013). Construction and characterization of nitrate and nitrite respiring *Pseudomonas putida* KT2440 strains for anoxic biotechnical applications. J Biotechnol.

[14] Nikel PI, de Lorenzo V (2013). Engineering an anaerobic metabolic regime in *Pseudomonas putida* KT2440 for the anoxic biodegradation of 1,3-dichloroprop-1-ene. Metab Eng.

[15] Schmitz S, Nies S, Wierckx N, Blank LM, Rosenbaum MA (2015). Engineering mediator-based electroactivity in the obligate aerobic bacterium *Pseudomonas putida* KT2440. Microbial Physiol Metab.

[16] Lai B, Yu S, Bernhardt PV, Rabaey K, Virdis B, Kromer JO (2016). Anoxic metabolism and biochemical production in *Pseudomonas putida* F1 driven by a bioelectrochemical system. Biotechnol Biofuels.

[17] Wu M, Guina T, Brittnacher M, Nguyen H, Eng J, Miller SI (2005). The *Pseudomonas aeruginosa* proteome during anaerobic growth. J Bacteriol.

[18] Glasser NR, Kern SE, Newman DK (2014). Phenazine redox cycling enhances anaerobic survival in *Pseudomonas aeruginosa* by facilitating generation of ATP and a proton-motive force. Mol Microbiol.

[19] Eschbach M, Schreiber K, Trunk K, Buer J, Jahn D, Schobert M (2004). Long-term anaerobic survival of the opportunistic pathogen *Pseudomonas aeruginosa* via pyruvate fermentation. J Bacteriol.

[20] Trunk K, Benkert B, Quack N, Munch R, Scheer M, Garbe J (2010). Anaerobic adaptation in *Pseudomonas aeruginosa*: definition of the Anr and Dnr regulons. Environ Microbiol.

[21] Hesse C (2018). Genome-based evolutionary history of pseudomonas spp. Environ Microbiol.

[22] Petzold CJ, Chan LJG, Nhan M, Adams PD (2015). Analytics for metabolic engineering. Front Bioengin Biotechnol.

[23] Kampers LFC, van Heck RGA, Donati S, Saccenti E, Volkers RJM, Schaap PJ (2019). *In silico*-guided engineering of *Pseudomonas putida* towards growth under micro-oxic conditions. Microb Cell Factories.

[24] Koehorst JJ, Dam JCJ, Heck RGA, Saccenti E, Santos VAPM, Suarez-Diez M (2016). Comparison of 432 *Pseudomonas* strains through integration of genomic, functional, metabolic and expression data. Sci Rep.

[25] Dotsch A, Schniederjans M, Khaledi A, Hornischer K, Schulz S, Bielecka A (2015). The *Pseudomonas aeruginosa* transcriptional landscape is shaped by environmental heterogeneity and genetic variation. mBio.

[26] Hartmans S, Smits JP, van der Werf MJ, Volkering F, de Bont JAM (1989). Metabolism of styrene oxide and 2-Phenylethanol in the styrene-degrading Xanthobacter strain 124x. Appl Environ Microbiol.

[27] Reddy TB, Thomas AD, Stamatis D, Bertsch J, Isbandi M, Jansson J (2014). The genomes online database (gold) v. 5: a metadata management system based on a four level (meta) genome project classification. Nucleic Acids Res.

[28] Amid C, Alako BTF, Balavenkataraman Kadhirvelu V, Burdett T, Burgin J, Fan J (2019). The European nucleotide archive in 2019. Nucleic Acids Res.

[29] Koehorst JJ, van Dam JCJ, Saccenti E, Martins dos Santos VAP, Suarez-Diez M, Schaap PJ (2018). SAPP: functional genome annotation and analysis through a semantic framework using FAIR principles. Bioinformatics.

[30] Hyatt D, Chen G-L, LoCascio PF, Land ML, Larimer FW, Hauser LJ (2010). Prodigal: prokaryotic gene recognition and translation initiation site identification. BMC Bioinformatics.

[31] Jones P, Binns D, Chang H-Y, Fraser M, Li W, McAnulla C (2014). InterProScan 5: genome-scale protein function classification. Bioinformatics.

[32] El-Gebali S, Mistry J, Bateman A, Eddy SR, Luciani A, Potter SC (2019). The pfam protein families database in 2019. Nucleic Acids Res.

[33] Koehorst JJ, Saccenti E, Schaap PJ, dos Santos VAM, Suarez-Diez M (2016). Protein domain architectures provide a fast, efficient and scalable alternative to sequence-based methods for comparative functional genomics. F1000Research.

[34] Anders S, Huber W (2012). Differential expression of rna-seq data at the gene level–the deseq package.

[35] Nogales J, Gudmundsson S, Duque E, Ramos JL, Palsson BO (2017). Expanding the computable Reactome in *Pseudomonas putida* reveals metabolic cycles providing robustness. bioRxiv.

[36] Nikel PI (2015). Pseudomonas putida KT2440 strain metabolizes glucose through a cycle formed by enzymes of the Entner-Doudoroff, Embden-Meyerhof-Parnas, and pentose phosphate pathways. J Biol Chem.

[37] Carmona M, Zamarro MT, Bl’azquez B, Durante-Rodr’ıguez G, Ju’arez JF, Valderrama JA (2009). Anaerobic catabolism of aromatic compounds: a genetic and genomic view. Microbiol Mol Biol Rev.

[38] Kovacs A, Rakhely G, Balogh J, Maroti G, Fulop A, Kovacs K (2005). Anaerobic regulation of hydrogenase transcription in different bacteria.

[39] Peters JW, Schut GJ, Boyd ES, Mulder DW, Shepard EM, Broderick JB (2015). [FeFe] and [NiFe]-hydrogenase diversity, mechanism, and maturation. Biochimica et Biophys Acta (BBA) Mol Cell Res.

[40] Crespo A, Blanco-Cabra N, Torrents E (2018). Aerobic vitamin B12 biosynthesis is essential for *Pseudomonas aeruginosa* class II Ribonucleotide Reductase activity during planktonic and biofilm growth. Front Microbiol.

[41] Fang H, Kang J, Zhang D (2017). Microbial production of vitamin B12: a review and future perspectives. Microb Cell Factories.

[42] Moore SJ, Lawrence AD, Biedendieck R, Deery E, Frank S, Howard MJ (2013). Elucidation of the anaerobic pathway for the corrin component of cobalamin (vitamin b12). Proc Natl Acad Sci.

[43] Roth JR, Lawrence JG, Rubenfield M, Kieffer-Higgins S, Church GM (1993). Characterization of the cobalamin (vitamin B12) biosynthetic genes of salmonella typhimurium. J Bacteriol.

[44] Richts B, Rosenberg J, Commichau FM (2019). A survey of Pyridoxal 5-phosphate-dependent proteins in the gram-positive model bacterium Bacillus subtilis. Front Mol Biosci.

[45] Wissenbach U, Kroger A, Unden G (1990). The specific functions of menaquinone and demethylmenaquinone in anaerobic respiration with fumarate, dimethylsulfoxide, trimethylamine N-oxide and nitrate by *Escherichia coli*. Arch Microbiol.

[46] Domrose A, Weihmann R, Thies S, Jaeger K-E, Drepper T, Loeschcke A (2017). Rapid generation of recombinant *Pseudomonas putida* secondary metabolite producers using ytrex. Synthetic Syst Biotechnol.

[47] Hutchison CA, Chuang R-Y, Noskov VN, Assad-Garcia N, Deerinck TJ, Ellisman MH (2016). Design and synthesis of a minimal bacterial genome. Science.

[48] Hong Enriquez RP, Do TN (2012). Bioavailability of metal ions and evolutionary adaptation. Life.

[49] Hatayama K, Kawai S, Shoun H, Ueda Y, Nakamura A (2005). *Pseudomonas* azotifigens sp. nov., a novel nitrogen-fixing bacterium isolated from a compost pile. Int J Syst Evol Microbiol.

[50] Yumoto I, Yamazaki K, Hishinuma M, Nodasaka Y, Suemori A, Nakajima K (2001). *Pseudomonas* alcaliphila sp. nov., a novel facultatively psychrophilic alkaliphile isolated from seawater. Int J Syst Evol Microbiol.

[51] Pitcher RS, Watmough NJ (2004). The bacterial cytochrome cbb3 oxidases. Biochimica et Biophysica Acta (BBA) Bioenergetics.

[52] Nizam SA, Shimizu K (2008). Effects of arca and arcb genes knockout on the metabolism in *Escherichia coli* under anaerobic and microaerobic conditions. Biochem Eng J.

[53] Nikel PI (2009). Metabolic flux analysis of Escherichia coli creB and arcA mutants reveals shared control of carbon catabolism under microaerobic growth conditions. J Bacteriol.

[54] Shalel-Levanon S, San K-Y, Bennett GN (2005). Effect of ArcA and FNR on the expression of genes related to the oxygen regulation and the glycolysis pathway in Escherichia coli under microaerobic growth conditions. Biotechnol Bioeng.

[55] Zhu J (2006). Effect of the global redox sensing/regulation networks on Escherichia coli and metabolic flux distribution based on C-13 labeling experiments. Metab Eng.

