# Supplementary figures and images for: A metabolic and physiological design study of Pseudomonas putida KT2440 capable of anaerobic respiration
Source: BMC Microbiol. 2021 Jan 6;21:9. doi: 10.1186/s12866-020-02058-1 (PMC7789669; doi:10.1186/s12866-020-02058-1)

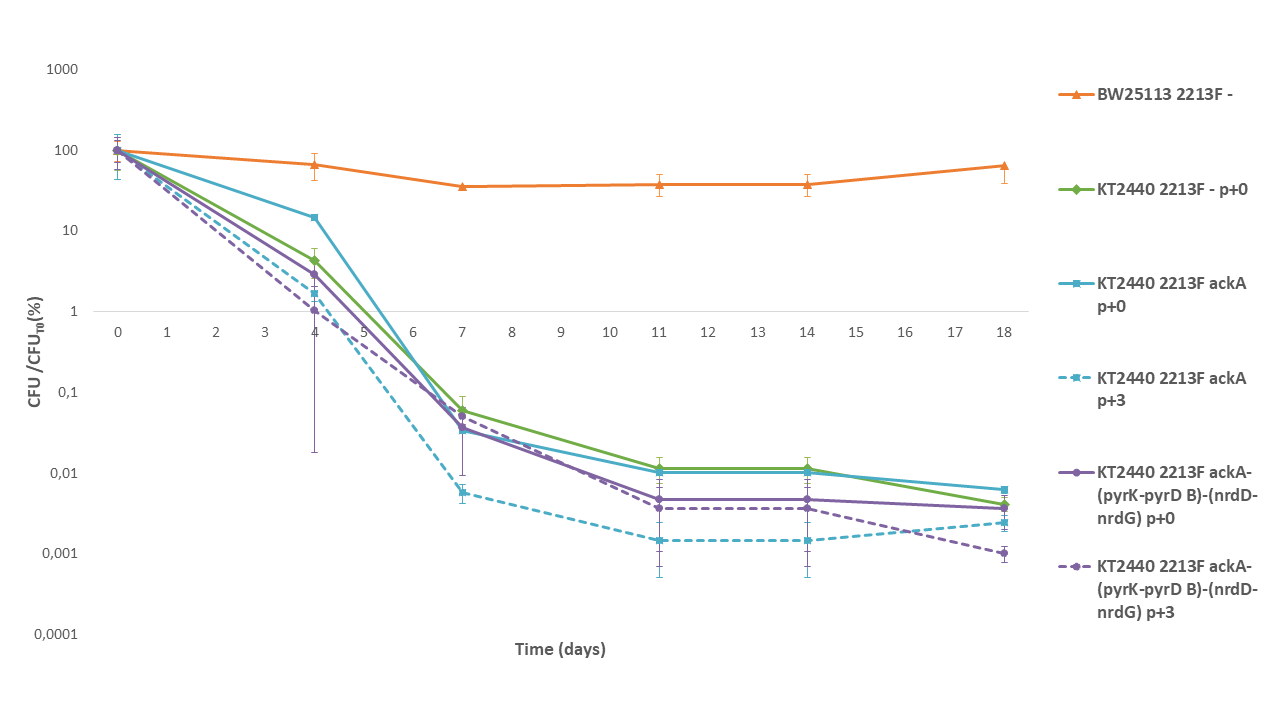

Supplement: Supplementary file 2 — Additional file 2: Figure S1. Anoxic survival of P. putida KT2440 transformed strains, grown on De Bont minimal medium with gluconic acid as sole carbon source and kanamycin. The headspace was flushed from oxygen with nitrogen. Survival under anoxic conditions was determined by comparing the number of colony forming units (CFU) over time with the number of CFU at T0. Escherichia coli BW25113 was used as positive control and Pseudomonas putida KT2440 with an empty plasmid (pS2213 -) was used as a negative control. Tested strains were Pseudomonas putida KT2440 with acetate kinase (pS2213 ackA) unpassed (p + 0) or passed three consecutive times over oxygen gradients (p + 3), and Pseudomonas putida KT2440 with acetate kinase, dihydroororotate dehydrogenase and ribonucleotide triphosphate reductase type II (pS2213 ackA-(pyrK-pyrD B)-(nrdD-nrdG) unpassed (p + 0) or passed three consecutive times over oxygen gradients (p + 3). [file 12866_2020_2058_MOESM2_ESM.tif]

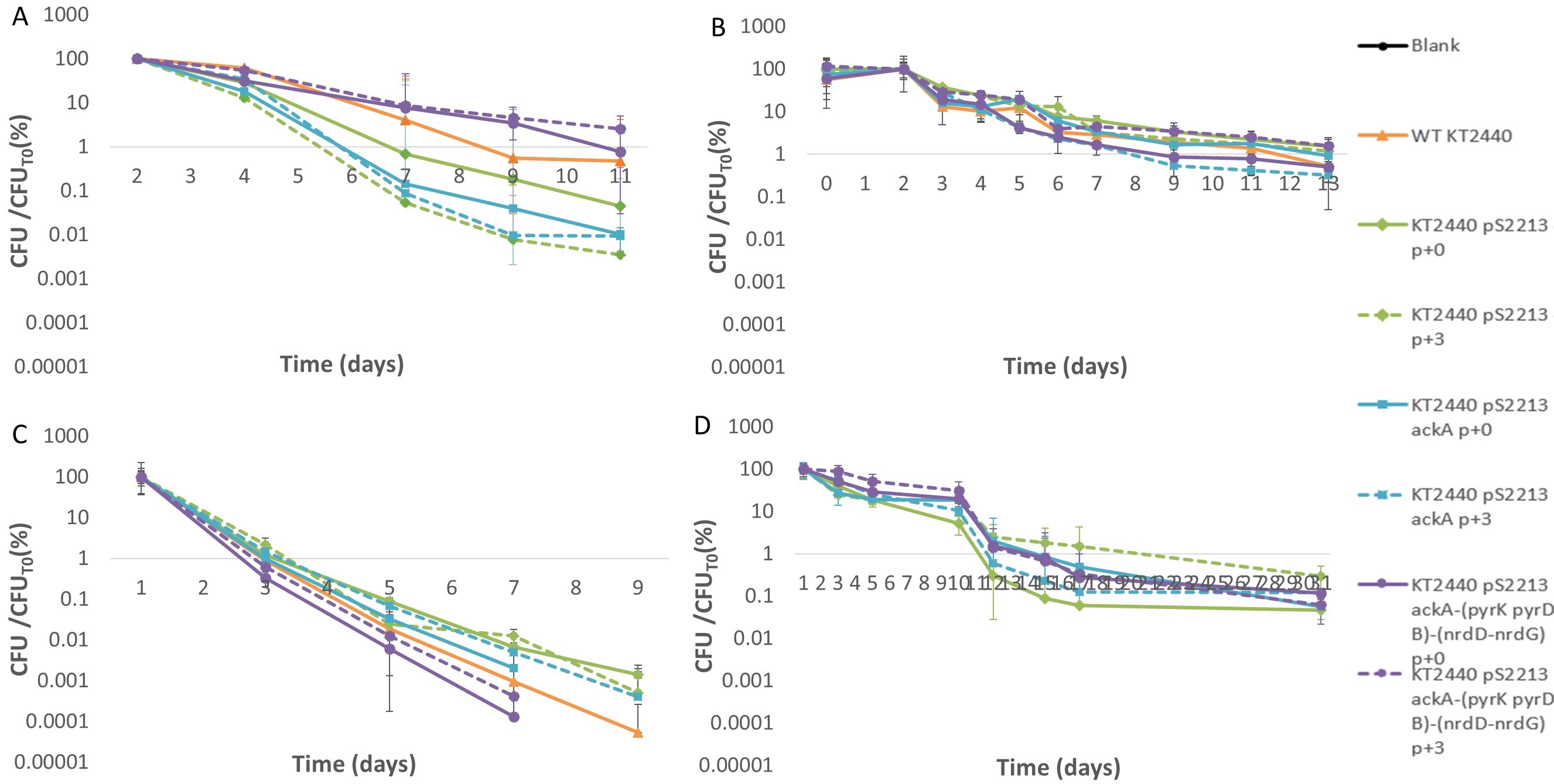

Supplement: Supplementary file 3 — Additional file 3: Figure S2. Survival experiment of P. putida KT2440 under anoxic conditions. The CFU determination of Pseudomonas putida KT2440 with an empty plasmid (pS2213 -), acetate kinase (pS2213 ackA) or acetate kinase, dihydroororotate dehydrogenase and ribonucleotide triphosphate reductase type II (pS2213 ackA-(pyrK-pyrD B)-(nrdD-nrdG) unpassed (p + 0) or passed three consecutive times over oxygen gradients (p + 3) survival under anoxic conditions. The experiment was repeated independently six times. All figures share the same legend. (A) Experiment 1 (B) Experiment 2 (C) Experiment 3 (D) Experiment 4. [file 12866_2020_2058_MOESM3_ESM.jpg]

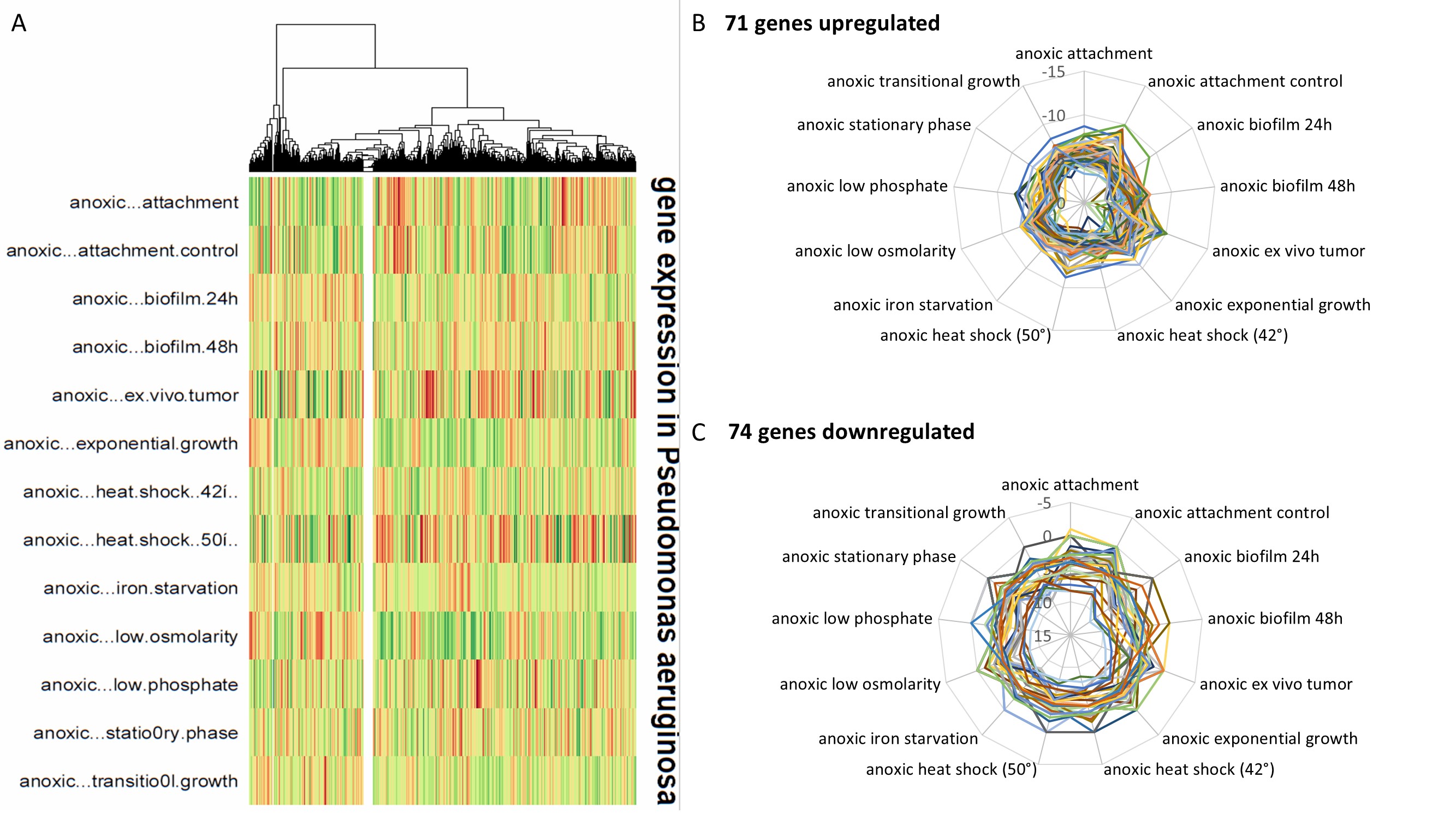

Supplement: Supplementary file 4 — Additional file 4: Figure S3. Transcriptomics of Pseudomonas aeruginosa PA01 in 15 different conditions. (A) Heatmap of up (green) or downregulation (red) of all genes per condition. (B) All upregulated genes per condition. (C) All downregulated genes per condition. [file 12866_2020_2058_MOESM4_ESM.jpg]

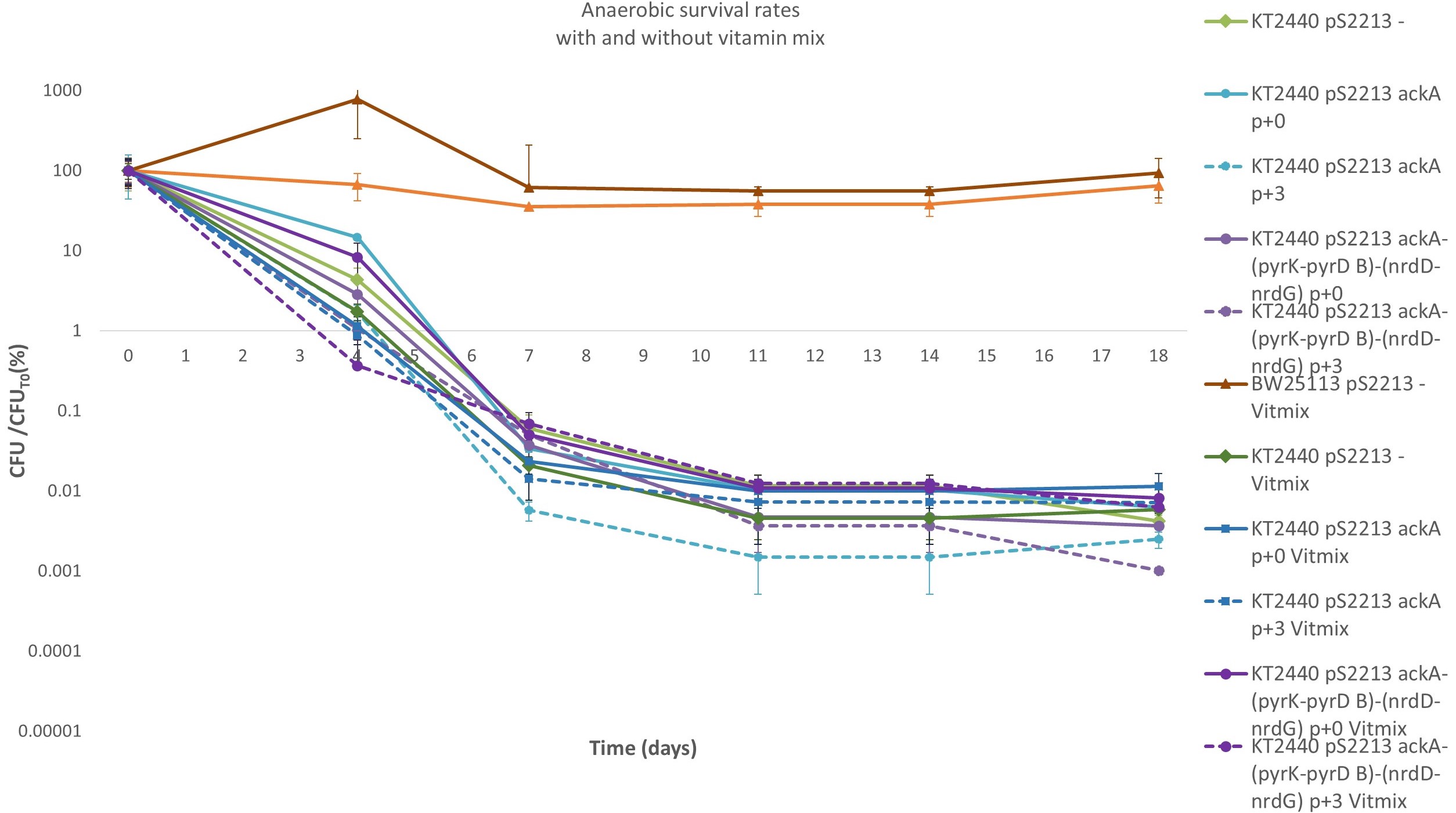

Supplement: Supplementary file 5 — Additional file 5: Figure S4. Survival experiment of P. putida KT2440 under anoxic conditions. The CFU determination of Pseudomonas putida KT2440 with an empty plasmid (pS2213 -), acetate kinase (pS2213 ackA) or acetate kinase, dihydroororotate dehydrogenase and ribonucleotide triphosphate reductase type II (pS2213 ackA-(pyrK-pyrD B)-(nrdD-nrdG) unpassed (p + 0) or passed three consecutive times over oxygen gradients (p + 3) with or without vitamin mix. [file 12866_2020_2058_MOESM5_ESM.jpg]

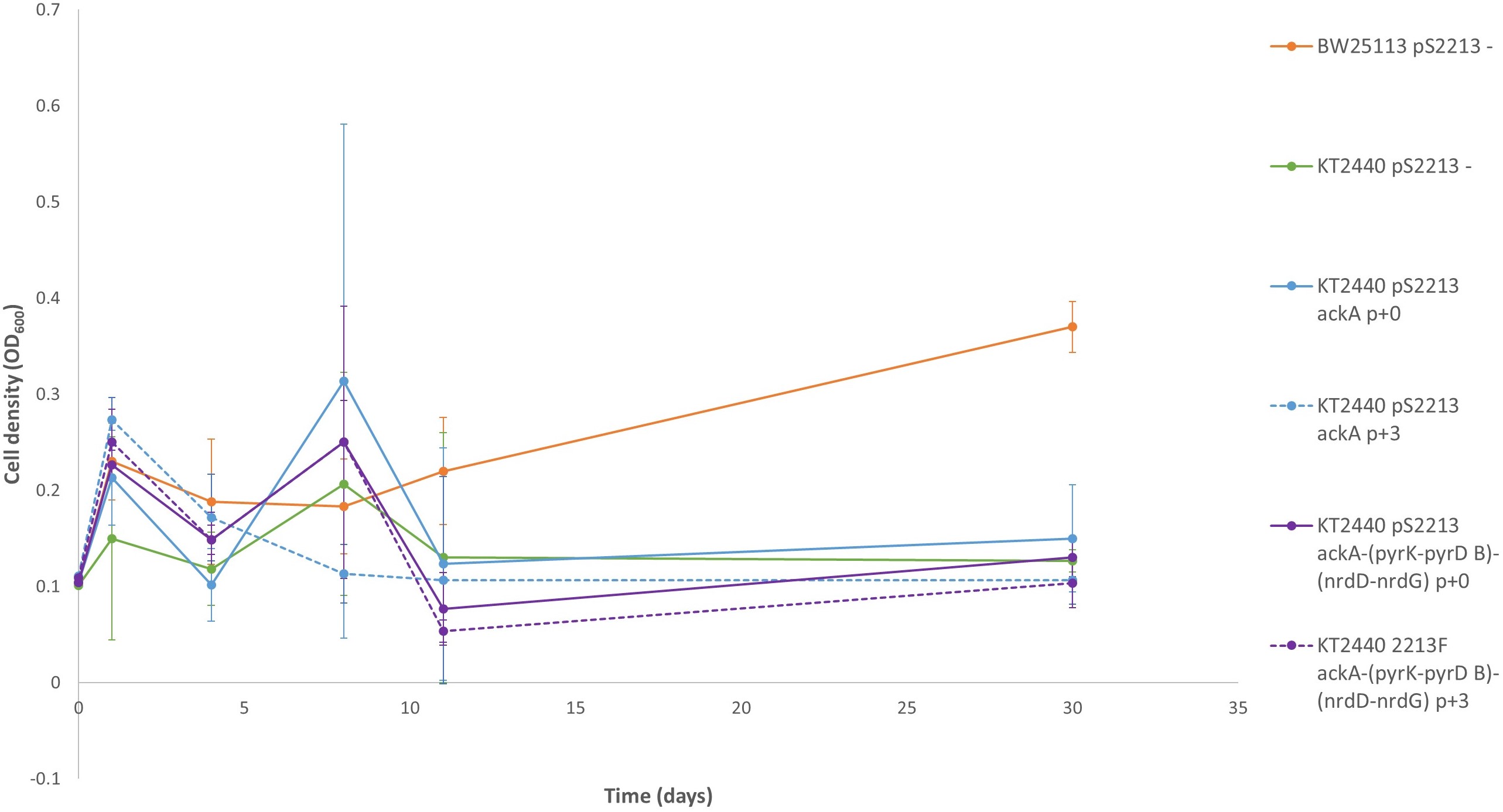

Supplement: Supplementary file 6 — Additional file 6: Figure S5. Growth experiment of P. putida KT2440 under anoxic conditions. The OD600 determination of Pseudomonas putida KT2440 with an empty plasmid (pS2213 -), acetate kinase (pS2213 ackA) or acetate kinase, dihydroororotate dehydrogenase and ribonucleotide triphosphate reductase type II (pS2213 ackA-(pyrK-pyrD B)-(nrdD-nrdG) unpassed (p + 0) or passed three consecutive times over oxygen gradients (p + 3) with vitamin mix. [file 12866_2020_2058_MOESM6_ESM.jpg]
