# Supplementary material for: A metabolic and physiological design study of Pseudomonas putida KT2440 capable of anaerobic respiration
Source: BMC Microbiol. 2021 Jan 6;21:9. doi: 10.1186/s12866-020-02058-1 (PMC7789669; doi:10.1186/s12866-020-02058-1)
Supplement: Supplementary file 18 — Additional file 18. Potential roles of Dau, Nif, Rnf, RhfH operons in anaerobic respiration. [file 12866_2020_2058_MOESM18_ESM.pdf]

Kampers et al., Additional file 18

Genes of the Dau Nif, RhfH and Rnf, protein families are required for energy conservation, cofactor biosynthesis, amino acid biosynthesis, nitrogen metabolism, nitrate-, nitrite- and nitrogen transport. Similar to the work by Steen *et al.,* these genes were considered a necessary addition in pursuit of a facultative anaerobic lifestyle.

FAD-dependent catabolic D-arginine dehydrogenase DauA, NAD(P)H-dependent anabolic L-arginine dehydrogenase, DauB and the transcriptional regulator of the dauBAR operon, DauR, are required for (regulation of) amino acid biosynthesis and metabolism under anaerobic conditions.

Nif is a hydrogenase part of the NIR/NAR operon, and is responsible for the reversible oxidation of molecular hydrogen and plays a vital role in anaerobic metabolism. Hydrogenases catalyse the reversible oxidation of molecular hydrogen and play a vital role in anaerobic metabolism. Metal-containing hydrogenases are subdivided into three classes: Fe ('iron only') hydrogenases; Ni-Fe hydrogenases; and Ni-Fe-Se hydrogenases [PUBMED:3078655]. Hydrogen oxidation is coupled to the reduction of electron acceptors (such as oxygen, nitrate, sulphate, carbon dioxide and fumarate), whereas proton reduction (hydrogen evolution) is essential in pyruvate fermentation or in the disposal of excess electrons. The Ni-Fe hydrogenases, when isolated, are found to catalyse both hydrogen evolution and uptake, with low-potential multihaem cytochromes, such as cytochrome c3, acting as either electron donors or acceptors, depending on their oxidation state. Both periplasmic (soluble) and membrane-bound hydrogenases are known.

Rnf is a family of integral membrane proteins including Rhodobacter-specific nitrogen fixation (rnf) proteins RnfA and RnfE [1] and Na+-translocating NADH:ubiquinone oxidoreductase (Na+-NQR) subunits NqrD and NqrE, and is considered vital in the NIR/NAR operon for nitrogen fixation.

A member of the RnfH family of the ubiquitin superfamily strongly co-occur in two distinct gene neighborhood contexts. In the first context it is associated with a START domain protein, a membrane protein SmpA and the transfer mRNA binding protein SmpB. This association suggests a possible role in the SmpB-tmRNA-based tagging and degradation system of bacteria, which is interesting given that other members of the ubiquitin system are analogously involved in protein-tagging and degradation across eukaryotes and various prokaryotes. In the second context the RnfH genes are present is in a membrane associated complex involved in transporting electrons for various reductive reactions such as nitrogen fixation. In this case, the latter could be true, as RnfH is included towards transporting electrons for various reductive reactions such as nitrogen fixation.

As Steen *et al.* already reported, the incorporation of either the NIR/NAR or the NOR operon proved to be insufficient to achieve an anaerobic lifestyle, and these genes came up in each tried method to differentiate between aerobic from anaerobic species, these genes were incorporated in the design.

Ureohydrolases such as Arg1, SpeB, HutG and Pah fulfil important roles in arginine/agmatine metabolism, the urea cycle, histidine degradation, and other pathways. Arginase, which catalyses the conversion of arginine to urea and ornithine, is one of the five members of the urea cycle enzymes that convert ammonia to urea as the principal product of nitrogen excretion. The ureohydrolases were preserved as they have a clear function within the ammonification process and were preserved throughout the anaerobic species.
